# Cardiopulmonary arrest in primary care clinics: more holes than cheese: a survey of the knowledge and attitudes of primary care physicians regarding resuscitation

**DOI:** 10.1186/s13584-017-0148-1

**Published:** 2017-06-10

**Authors:** Sharon Einav, Oren Wacht, Nechama Kaufman, Eliezer Alkalay

**Affiliations:** 1 Surgical Intensive Care, Shaare Zedek Medical Center, and Anesthesia and Intensive Care Medicine, Hebrew University-Hadassah Faculty of Medicine, POB 3235, Beyt 12, Jerusalem, 91031 Israel; 2 Department of Emergency Medicine, Faculty of Health Sciences, Ben Gurion University of the Negev, Beer-Sheba, Israel; 3 Departments of Emergency Medicine and Intensive Care Unit, Shaare Zedek Medical Centre, Jerusalem, Israel; 4 Herut-Mishmeret Family Medicine Clinic, Sharon-Shomron section of the Clalit Healthcare Services, associated with the Sackler Faculty of Medicine, Tel-Aviv University, Tel-Aviv-Yafo, Israel

**Keywords:** Cardiopulmonary resuscitation, Delivery of Health Care, Quality of Health Care, Primary Health Care, Cardiopulmonary Arrest

## Abstract

**Background:**

Patients experiencing pre-arrest symptoms may first refer to their primary care physician. The study's aim was to determine the likelihood that a patient undergoing out-of-hospital cardiac arrest will receive appropriate resuscitation efforts in a primary care clinic in a country with a directive that clinics maintain resuscitation equipment and physicians undergo periodic resuscitation training.

**Methods:**

An anonymous, 23-question online cross-sectional survey was created and administered to primary care physicians working in community clinics (10/1/2015-5/3/2015). Recruitment was accomplished by posting a link to the survey to all physicians listed as registered Society of Family Medicine members and in other online forums dedicated to residents and board-certified specialists in family medicine in Israel. The primary outcome measure was the proportion of respondents whose responses indicate that they fulfill all conditions for performing resuscitation.

**Results:**

Of approximately 2400 potential respondents, 185 replied to the survey; the study's findings should be viewed as preliminary. Respondents' characteristics were generally similar to those of the study population, but respondents had a higher rate of family medicine specialists.

Respondents were mostly female (*n* = 108, 58%) Israeli graduates who have practiced medicine for > 10 years (72%, *n* = 134). 55% (*n* = 101) had undergone basic life support (BLS) training within < 2 years.

Although just 5% (*n* = 10) estimated call-to-Emergency Medical Service (EMS) arrival time to their clinic to be <5 min, only 64% (*n* = 119) knew the telephone number for summoning EMS. Most confirmed the existence of a resuscitation cart in their clinic (85%, *n* = 157); 68% confirmed the presence of a defibrillator (*n* = 126). Most respondents were aware of the location of the defibrillator in their clinic (67%, *n* = 123), stated its accessibility during working hours (63%, *n* = 116), and 56% (*n* = 103) knew how to use it. Only 28% of the questionnaires indicated that all requirements for mounting an effective BLS response had been fulfilled.

**Conclusions:**

The study suggests that many primary care clinics are under-equipped and their physicians are under-prepared to initiate life-saving services. Steps must be taken to rectify this situation. In addition, to develop more reliable estimates of the phenomena reported in this preliminary study, these issues should be re-examined in the context of a high response rate physician survey.

## Background

Survival rates from out-of-hospital cardiac arrest (OHCA) are highly variable, ranging from 3.0% to 16.3% [1]. Immediate recognition and delivery of high quality basic life support (BLS) is one of the most crucial factors in neurologically intact survival [2]. Many OHCA patients receive initial treatment by bystanders or by emergency medical services (EMS) [3]; however, some patients experiencing early symptoms may seek help from their primary physician/urgent care center and/or collapse in the clinic itself [4–6]. In the United States, 2% of persons who survived OHCA were treated initially in a clinic [3].

The clinic physician’s response to a cardiac arrest in their clinic depends on multiple factors, including training and the availability of equipment and supplies. If the response is appropriate, survival may be quite high; survival of patients treated by primary care physicians equipped with defibrillators has been reported to be as high as 34% [7]. However, in many countries, there is limited standardization in either resuscitation training or mandated equipment within medical facilities/clinics that are not hospitals or EMSs. The challenge of maintaining competence and confidence in critical clinical skills used infrequently is now gaining recognition [8, 9].

The present study examined the likelihood that a patient would receive an appropriate resuscitation attempt in their primary care clinic by studying clinic preparedness for performing BLS. Our baseline hypothesis was that most clinics are underprepared for performing cardiopulmonary resuscitation (CPR).

## Methods

After Shaare Zedek Medical Centre's Institutional Review Board (IRB) approval (number P6.15), a cross-sectional survey was administered to physicians working in community clinics. In accordance with IRB demand, informed consent for use of the survey data for research purposes was provided by participants directly within the survey.

### Clinical setting

The National Israeli Health Insurance Law determines that all medical treatment should be both equal and equally accessible to all citizens of the state. Membership in one of the four Health Maintenance Organizations (HMOs) is mandatory, as is an income-graduated healthcare tax. The Treasury covers the difference between the actual cost of service provided and the income generated by paying members, thus ensuring that insurance coverage is not related to income. Israel provides a uniform basic healthcare package which covers the cost of lifesaving services, as CPR is considered the default procedure for treatment of OHCA. EMS guidelines are that all OHCA patients should undergo resuscitation attempts unless a valid Do Not Attempt Resuscitation order exists or irrevocable signs of death (e.g. rigor mortis, decapitation, dependent lividity) are determined by the paramedic on location.

Physicians staffing either private or HMO clinics are comprised of general practitioners (up to 50%) as well as physicians who are board-certified in such fields as Family Medicine, Pediatrics, Internal Medicine and Geriatric Medicine. In 2012, the Israeli Ministry of Health issued the first directive addressing CPR in primary care clinics. This directive instructs physicians working in such clinics to undergo periodic training. It also mandates that clinics serving >3000 insurees have a resuscitation cart fully equipped for providing ALS (advanced life support), including (at least) an automatic external defibrillator, while absolving small clinics (defined as those serving <3000 insurees) from the need to have any kind of defibrillator (http://www.health.gov.il/hozer/mr04_2012.pdf).

Israel has a three-tiered National EMS. All calls to the number 101 from any telephone in the country are routed directly to a central dispatch center. All available responders in the area (regardless of tier) are identified using an automated Geographic Information System locator and dispatched to the location of the arrest. EMS response times to a call coming from a primary care clinic will thus depend on the location of the clinic and the proximity of vehicles in the area at the time of the call. Similar to other places in the world, ambulance coverage also depends on population density, distance and traffic. Response times are usually >4 min – the threshold for significant time-sensitive survival [10]. Patient prognosis is therefore highly dependent on the actions of the bystanders witnessing the arrest.

### Study population

Practicing primary care physicians.

### Primary outcome measure

The proportion of respondents whose responses indicate that they would be capable of delivering quality Basic Life Support prior to EMS arrival.

In order to achieve this aim, we surveyed our sample population regarding the presence of prerequisite conditions for performing resuscitation (knowledge, training, willingness and equipment). The assumption was that the initial call for EMS assistance would be placed by another person on location while the physician provided BLS.

### Study tool

The survey was generated through collaboration between an expert in family medicine (working in a representative primary care clinic) (EA), a representative of the EMS (OW) and an expert in intensive care, resuscitation and research (SE), thus promoting both content and expert validity of the study tool. Following multi-disciplinary discussions, a questionnaire comprised of 23 questions was created for the purpose of this study. The clarity of the questions and their relevance was validated by two external consultants.

The questionnaire included no personal identifiers. Details regarding respondent demographics and clinic characteristics were constructed so as to provide data relevant to the study without disclosing personal information, thus ensuring confidentiality and anonymity.

The questionnaire included closed questions regarding: the type and location of the clinic and the population it serves, the presence, location and accessibility of resuscitation equipment in the clinic and the training, experience and willingness of the respondent to perform cardiopulmonary resuscitation.

### Survey method

An online survey was created using the Google survey tool. The link to the survey was posted in: the email list of all family doctors registered as members of the Society of Family Medicine through the Israel Medical Association, the local Facebook forum of "Residents and young experts in family medicine" and the online forum of "Primary physicians in the community- residents and experts". Posts were accompanied by an explanation regarding the importance of the survey. Candidates for participation received two posts using each method, with a time lag approximating two weeks between the first and second post. We estimate that approximately 2400 candidates were approached using the combination of these methods. Data were collected between 10-Jan-2015 and 5-March-2015. All surveys were self-administered. There was no follow-up on initial non-responders.

### Statistical analysis

Data were converted to an SPSS database (IBM SPSS Statistics for Windows, Version 21.0. Armonk, NY: IBM Corp). Analysis was performed using the same software. Missing responses to specific questions in the completed questionnaires were coded as missing. Only 1% (*n* = 2) of the questionnaires were excluded for lack of data (<90% complete responses). Statistical analysis included descriptive statistics (e.g. number and percent of respondents who chose each response option). Percentages were calculated from the total number of respondents, including those missing a response. We used either the chi-square test or the Fisher's exact test for comparisons after studying whether variable distribution was normal. A *p*-value <0.05 was considered significant.

## Results

Overall, 185 primary care physicians responded to the questionnaire. Most respondents were female physicians (*n* = 108, 58%) who had graduated from medical school in Israel and had been practicing medicine for more than 10 years (72%, *n* = 134) (Table 1). In Table 2, the characteristics of the physicians who responded to the questionnaire are presented alongside the characteristics of the general population of Israeli physicians working in primary care [11, 12]. The two groups are similar with regard to most of the characteristics examined. However, the proportion of physicians with formal training in family medicine was somewhat higher; this is unsurprising given that the platforms for promoting the survey most likely include a higher number of registrants with formal training.

**Table 1:** Respondent characteristics

Characteristic	% (*n* = 185)
*Sex*	---
Female	58.4% (*n* = 108)
Male	37.8% (*n* = 70)
*Years since graduation from medical school*	---
<5	6.5% (*n* = 12)
5-10	18.5% (*n* = 35)
10-20	31.9% (*n* = 59)
>20	40.5% (*n* = 75)
*Country of graduation from medical school*	---
Israel	57.8% (*n* = 107)
Elsewhere	41.1% (*n* = 76)
*Medical Specialty*	---
Board certified family care physician	67.9% (*n* = 129)
Other board certified expertise	15.1% (*n* = 28)
Resident	10.3% (*n* = 19)
*Location*	---
Urban	72.4% (*n* = 134)
Rural	27.6% (*n* = 51)
*BLS training*	---
Yes	98.3% (*n* = 180)
No	1.6% (*n* = 3)
*Time of BLS training*	---
<2 Years	54.6% (*n* = 101)
2-5 Years	34.6% (*n* = 64)
>5 Years	7.6% (*n* = 14)

Missing data: Sex *n* = 7, Years since graduation *n* = 4, Country of graduation *n* = 2, Specialty *n* = 9, BLS training *n* = 2, Time of BLS training *n* = 6

**Table 2:** Demographics of the study population compared to the demographics of both experts in Family Medicine and physicians working in community clinics as primary care physicians. Data culled from Israeli Ministry of Health reports (refs [11, 12])

	Study population (*n* = 185)	Israeli experts in Family Medicine (*n* = 1901)	Israeli physicians working as primary care physicians (*n* = 4627)
Gender	56.9% Female	56% Female	45% Female
Age	Average 45.4 ± 8.8 (Range 30-69)	50.4% < 50^a^	37% < 50^a^
Country of Birth	53.5% Israel	ND	34.5% Israel
Country of MD training	62.8% Israel	ND	35.7% Israel
	17.8% Former USSR		41.1% Former USSR
	11.6% Europe/America		24% Europe/America
Specialization	66.4% Family Medicine	Not relevant	32% Family Medicine
	16% Residents		44% Non-specialists
	17.6% Other specialization or non-specialists		24% Other specialization (approx. 50% of whom are specialists in Internal Medicine)
Form of employment	80% Employed	62% Employed	ND
	20% Self Employed	6% Self Employed	
		32% Both	
HMO	44.4% Clalit	ND	52% Clalit
	43% Maccabi		25% Maccabi
	11% Meuhedet		13.6% Meuhedet
	2.4% Leumit		9% Leumit
Place of employment	80% community clinic	79% community clinics	39% community clinics
	14% rural community clinic		54% hospitals
	3.2% IDF military clinics		
	2.4% mostly in hospitals		

^a^ age grouped by decade from 21-30 to 71+

### Clinic characteristics

Most of the respondents stated that they worked in an urban clinic (72%, *n* = 134), while only a minority worked in a smaller community clinic (27%, *n* = 51). Despite this, only 5% of the respondents (*n* = 10) stated that they estimated the call-to-arrival time of an EMS ambulance to their clinic in the event of a cardiac arrest would be <5 min, 52% (*n* = 97) estimated this would take 5-10 min and the rest estimated that arrival times would be >10 min (41%, *n* = 76).

### BLS training, call for help and willingness to perform CPR

**Fig. 1:**
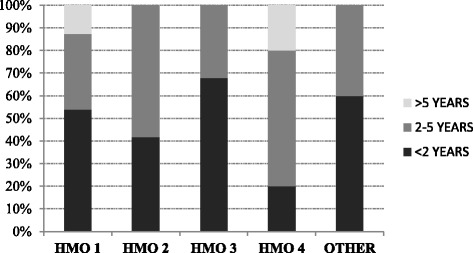
Physicians' most recent BLS training by HMO provider

Almost all the respondents had undergone BLS training (97%, 180/185); half within the last two years (55%, 101/185) and the rest at least 3-5 years ago (35%, 64/185). Although no difference was observed in the proportion of physicians who had undergone BLS training in the different HMOs, a borderline difference was observed in the time of most recent training, with training in the last two years ranging between 20% and 68% in the different HMOs (*p* = 0.048) (Fig. 1).

Less than two-thirds of the respondents (64%, 119/185) knew the telephone number needed to summon an ambulance from the National Israeli EMS. Respondents were almost unified in stating that they would be willing to provide BLS to a patient in their clinic if required to do so (95%, 175/185). Respondents who declared that they would not perform BLS were requested to provide a reason for their refusal. The main reason cited for inaction was physical disability (*n* = 6).

### Presence, location and accessibility of resuscitation equipment in the clinics

**Fig. 2:**
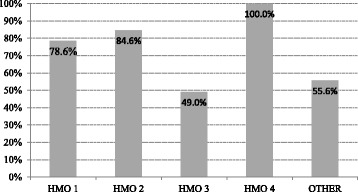
Presence of defibrillator by HMO provider

Respondents were asked whether there is a resuscitation cart in their clinic. Approximately 5 out of 6 responded positively (85%, 157/185), yet only two-thirds of the respondents declared the existence of a defibrillator in their clinic (68.1%, 126/185). A significant difference was observed between the HMOs regarding the presence of a defibrillator in their clinics (*p* = 0.001) (Fig. 2), however a defibrillator was equally likely to be present in an urban versus a rural clinic. Approximately 2 out of 3 respondents claimed to know the location of the defibrillator in their clinic (67%, 123/185) and declared that it is easily accessible (63%, 116/185). When asked whether they would know how to use a defibrillator, just over one-half of the respondents said they would (56%, 103/185); the proportion of positive responses to this question was unrelated to HMO affiliation. Finally, the respondents were asked whether their clinic is equipped to provide CPR to a child. Only about half responded positively (56%, 104/185), while most of the remaining respondents stated that they did not know (22%, 40/185).

### Cumulative reduction in the conditions required for effective resuscitation

**Fig. 3:**
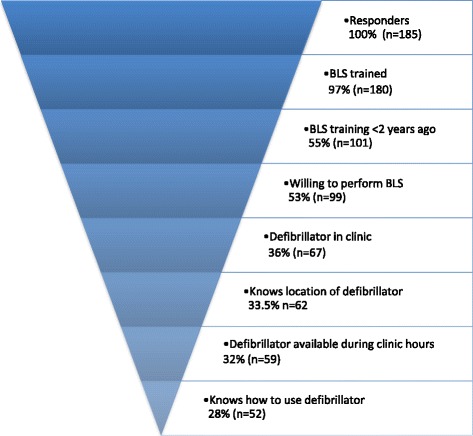
Cumulative reduction in the proportion of respondents that provided an appropriate response to all the components required to mount an effective resuscitation response. The assumption was that the initial call for EMS assistance would be placed by another person on location while the physician provides BLS

Figure 3 presents the cumulative reduction in the proportion of respondents that would be able to provide an appropriate response for each successive step of a resuscitation attempt, assuming that all prior steps have been fulfilled. Every step in the proper response to a cardiac arrest is sequential: for example, first a defibrillator must exist in the clinic, then the provider must know its location in order to access it, then the provider must be able to operate it, etc. In contrast to the descriptive statistics in the three previous subsections, in a cumulative reduction, if a certain number of respondents answered negatively at a given stage, this would mean that that number of respondents would be unable to proceed to the next step, thus allowing us to calculate the proportion of respondents that would be able to complete all steps in the required sequence. Therefore, based on the assumption that all the components of BLS must be met in order to mount an effective resuscitation response, no more than 28% of the respondents would be capable of providing an effective response to cardiac arrest if they were in the clinic with untrained laypersons.

## Discussion

The current paper suggests that many primary care clinics in Israel are woefully underprepared to provide effective CPR. In order to effectively respond to a cardiac arrest, all components must be present and aligned, but this often does not happen. In accident analysis, this mode of systems thinking is termed the "Swiss Cheese Model" [13]. In the current study, most physicians were willing to provide BLS but only half of them had undergone training within the time frame recommended for retaining resuscitation skills. Even so, there was no correlation between timely training and either the availability of a defibrillator or the physician's confidence in using it. Overall, almost three-quarters of the questionnaires demonstrated at least one potentially fatal missing link in the chain of survival.

Unfortunately, the response rate in our study was very low. Although the number of responses generated in this study was higher than that of previous studies on this issue [14, 15], questions remain regarding the representativeness of our sample. Our study population was approached through two online websites and a registry, thus pre-selection of younger primary physicians with a greater amount of training and greater motivation to remain "in the loop" is a possibility. Willingness to respond may have been driven by greater involvement or by pride in clinic organization (resulting in over-estimation of preparedness) or by physician realization that he/she is unprepared (resulting in under-estimation of preparedness). Non-response may have also been driven by a belief that the issue is irrelevant as the likelihood of being required to perform CPR in a clinic is very low (i.e. denial and a lack of interest). Thus the likelihood of a series of “correct” responses may in fact be significantly lower, as one would not likely prepare for an event that one believes unlikely to occur. Recall bias may also exist. However, such bias is unlikely to be systematic unless it is driven by the presence of a traumatic experience with CPR in the clinic. In summary, this survey could have been biased either way. Nevertheless, such findings should prompt more comprehensive investigation of this topic by the policy maker.

In the Jerusalem district study carried out in 2004-2010 [16, 17], 1.3% of cardiac arrests occurred in a primary care clinic (Einav S., unpublished data). Other countries have reported that between 6.1% to 13.8% of arrests occurred in the community [14, 18–20]. Since cardiac arrest in a healthcare clinic setting is clearly not a rare phenomenon, it seems appropriate that medical facilities be properly equipped and their staff be properly trained in the event of a resuscitation.

Calling for help is the first link in the chain of survival. Layperson knowledge of the telephone number to be called at the time of a medical emergency has been shown to be lacking in multiple studies [21–26]. Unnecessary delays caused by incorrect placement of a call results in a smaller proportion of cases presenting with Ventricular Fibrillation, demonstrating that this first link in the chain of survival – calling (the proper people) for help - is critical indeed [21]. We queried professionals who are usually assumed to know the EMS number, yet one-third of the physicians responding to our survey did not know the nation-wide emergency number to summon EMS. We found no prior study of the knowledge of outpatient clinic staff regarding this issue. Although during clinic working hours most likely either a nurse or a clerk would call the EMS, there is no guarantee that their knowledge is greater than that of the physicians surveyed. In fact, given the data from surveys quoted above, it may be less. This finding alone requires urgent intervention on a national scale; it also suggests the possibility of ongoing ignorance amongst the population with less medical awareness.

A simple and cheap solution to this problem could be enforcement of mandatory and standard signage in all clinics in a pre-specified and obvious location that is often observed (e.g. the entrance door next to the opening hours). The sign should include three instructions only: how to call for help ("Call number 101 from any telephone"), the location of the closest defibrillator ("If someone is with you send them to bring a defibrillator from XXXXX") and how to initiate basic life support ("Place your hands on the center of the chest and push at a rate of 100-120 per minute"). Concerns regarding the legalities of performing chest compression may be allayed by adding the wording of the local Good Samaritan law at the bottom of the sign in smaller lettering.

Delivery of quality chest compressions and timely defibrillation comprise the second and third links in the chain of resuscitation. However, the Ministry of Health directive regarding management of resuscitation in community clinics mandates that small clinics need not be equipped with a defibrillator (http://www.health.gov.il/hozer/mr04_2012.pdf), thus restricting the available treatment options in these facilities. Data collected from respondents did not include size of clinic so as to maintain anonymity, limiting our ability to derive meaning from the differences in the presence of defibrillators by HMO provider (Fig. 2). Be that as it may, one consideration in determining that not all primary clinics can be expected to own a defibrillator is based on their cost. The policy maker may have assumed that defibrillators are expensive when, in fact, an average AED currently costs about $1000 on e-bay. Another possible justification for leniency towards smaller clinics is the assumption that the EMS team will bring the first defibrillator in a timely manner. Most laypersons would expect the physician on location to take control of such an event should it occur in a clinic. This assumption is given additional validity by the presence of defibrillators in larger clinics and EMS arrival times that almost always exceed 4 min. Geographically, Israel is less disadvantaged than countries where research into the issues challenging remote rural medicine have been conducted [8, 9]. However, regardless of actual location, if for any reason practitioners do not have the ability to mount the basic response to a cardiac arrest, a clinic in a well-populated area may as well be in a remote inaccessible location.

Medical clinics are not the only places expected to have stationary defibrillators. Even before publication of the 2014 defibrillator law mandating that all public locations hosting more than 500 people daily must be equipped with a defibrillator [27], these devices could already be found in sports facilities, synagogues, schools, shopping centers and multiple other public locations. The law mandates that all such devices be registered in a national repository of data regarding the availability, location and maintenance of defibrillators. Enforcement of this law by the Ministry of Health would allow not only clinics but also all civilians access to the location of the nearest functional defibrillator at all times. While this may not be in the best interest of the EMS which has equipped all vehicles with AEDs, this should be considered necessary until such time as EMS arrival times improve.

The current study was conducted in a single country which may limit the generalizability of our findings. However, the publications that we did find on this topic supported our findings [14, 28]. Others too have demonstrated that primary care clinics may be insufficiently prepared for resuscitation. In a survey conducted among the workers of 141 health centres in Finland, only 18% of respondents considered resuscitation training in their health centre to be sufficient and systematic [28].

The initial response in the first few moments after cardiac arrest determines patient outcome. Only two interventions have been proven effective – early chest compression and defibrillation [29]. Both are simple and relatively cheap and neither requires advanced cardiac life support training or equipment. Optimising the basic response to cardiac arrest in primary care clinics is not a complex challenge, it is simply a question of priorities.

## Conclusions

Primary care clinics are the bedrock of community medicine. Cardiac arrests in these clinics are uncommon but life-threatening occurrences. A swift and appropriate response to these events is crucial for patient survival. This response includes physician knowledge, willingness and preparedness for performing basic CPR and defibrillation as appropriate. The current study suggests that many primary care clinics are underequipped and the physicians staffing them underprepared to provide such life-saving services. It is imperative that steps be taken to rectify this situation. In addition, to develop more reliable estimates of the phenomena reported in this preliminary study, these issues should be re-examined in the context of a high response rate physician survey.

## Abbreviations

ALS: Advanced life support
BLS: Basic life support
CPR: Cardiopulmonary resuscitation
EMS: Emergency medical services
HMO: Health Management Organization
IRB: Institutional Review Board
OHCA: Out-of-hospital Cardiac Arrest
